# AlGaN/GaN Metal Oxide Semiconductor High-Electron Mobility Transistors with Annealed TiO_2_ as Passivation and Dielectric Layers

**DOI:** 10.3390/mi14061183

**Published:** 2023-05-31

**Authors:** Yu-Shyan Lin, Chi-Che Lu

**Affiliations:** 1 Department of Materials Science and Engineering, National Dong Hwa University, Hualien 974301, Taiwan; 2 Department of Opto-Electronic Engineering, National Dong Hwa University, Hualien 974301, Taiwan

**Keywords:** MOS-HEMT, AlGaN, GaN, silicon substrate, TiO_2_, passivation, dielectric, MOCVD, anneal

## Abstract

This paper reports on improved AlGaN/GaN metal oxide semiconductor high-electron mobility transistors (MOS-HEMTs). TiO_2_ is used to form the dielectric and passivation layers. The TiO_2_ film is characterized using X-ray photoemission spectroscopy (XPS), Raman spectroscopy, and transmission electron microscopy (TEM). The quality of the gate oxide is improved by annealing at 300 °C in N_2_. Experimental results indicate that the annealed MOS structure effectively reduces the gate leakage current. The high performance of the annealed MOS-HEMTs and their stable operation at elevated temperatures up to 450 K is demonstrated. Furthermore, annealing improves their output power characteristics.

## 1. Introduction

High-electron mobility transistors (HEMTs) have been intensively investigated over a long period. GaN has the favorable properties of a big bandgap, an enhanced breakdown electric field, and high thermal conductivity. AlGaN/GaN is a key material system in fifth generation (5G) applications in the field of microwave communication [1,2,3,4]. An AlGaN/GaN heterostructure generates a high two-dimensional electron gas (2-DEG) by spontaneous and piezoelectric polarization effects [5,6].

Ibbetson et al. presented a surface donor model to explain the origin of 2-DEG in an AlGaN/GaN heterojunction [7]. Generally, 2-DEG electrons are formed when the thickness of the AlGaN layer is larger than the critical value. Polarization phenomena in the AlGaN/GaN structures generate surface states. The donor-like states (positively charged surfaces) can trap the negative charges and thereby generate a virtual gate, which reduces the 2-DEG concentration [5,6]. Moderate surface passivation can prevent the appearance of the virtual gate. Another issue associated with the AlGaN/GaN HEMT is the high leakage currents through the Schottky gate. Inserting a gate dielectric can significantly reduce the gate’s leakage. The use of many insulators with a high dielectric constant to reduce the leakage current and prevent the significantly negative shift of the threshold voltage has been investigated. Particular insulators have been intensively examined. They include the insulating dielectric materials AlO_X_ [8], Al_2_O_3_ [9,10,11,12,13,14,15], TiO_2_ [15,16,17,18], ZrO_2_ [19,20], SiN_X_ [21], and SiO_2_ [22,23,24]. TiO_2_ has high dielectric constants, making TiO_2_ an excellent insulating material for use in MOS devices. Little has been published on GaN-based MOS-HEMTs with a TiO_2_ insulating layer, about which much remains unknown.

The effects of high temperature and surface passivation on GaAs-based HEMTs have been extensively examined [25]. However, few studies of the potential of AlGaN/GaN metal oxide semiconductor high-electron mobility transistors (MOS-HEMTs) for high-temperature operation have been undertaken. In our previous report, the threshold voltage (*V*_th_), interface traps (*D*_it_), unity current gain cutoff frequency (*f_T_*), maximum frequency of oscillation (*f*_max_), and minimum noise figure (NF_min_) of the TiO_2_ AlGaN/GaN MOS-HEMTs were examined [17]. However, analyses of their constituent materials, their high-temperature output characteristics, and their power characteristics have not been discussed. Furthermore, to the best of our knowledge, no research has been performed on the power characteristics of TiO_2_ AlGaN/GaN MOS-HEMTs and their performance at high temperatures, which are worthy of investigation.

In this study, a material analysis of TiO_2_ is first conducted. X-ray photoemission spectroscopy (XPS) and Raman spectroscopy are used to characterize TiO_2_ films. Experimental results demonstrate that annealing improves the performance of MOS-HEMTs. The current–voltage characteristics of the MOS-HEMTs that are operated up to 450 K are measured.

## 2. Fabrication and Structure of Device

The epilayers of transistors were grown via low-pressure metal organic chemical vapor deposition (LP-MOCVD) on a silicon substrate. A typical AlGaN/GaN HEMT epitaxial structure that comprised of an undoped buffer layer, an undoped GaN layer (1.2 mm), an undoped Al_0.26_Ga_0.74_N top barrier layer (30 nm), and an undoped GaN cap layer (2 nm), was grown.

In order to generate the designed patterns, standard photolithography and lift-off techniques were used. First, the areas of mesa isolation were defined by inductively coupled plasma reactive ion etching (ICP-RIE), which is a means of dry etching. The purpose of mesa isolation is to reduce the leakage currents. Thermally evaporated Ni was used as the mesa etching mask. After the etching step, the Ni hard mask was removed completely using HNO_3_. Then, the source and drain contacts were formed by a Ti (10 nm)/Al (100 nm)/Au (50 nm) ohmic metal alloy. Finally, a Ni/Au (100/50 nm) gate metal stack was deposited without a recess or any extra dielectrics for the HEMT device. In this experiment, the device was spin-coated with positive photoresist (FH6400L) using a spinner. Ni has a high work function and so heightened the Schottky barrier. Au was used to prevent the oxidation of Ni.

**Figure 1:**
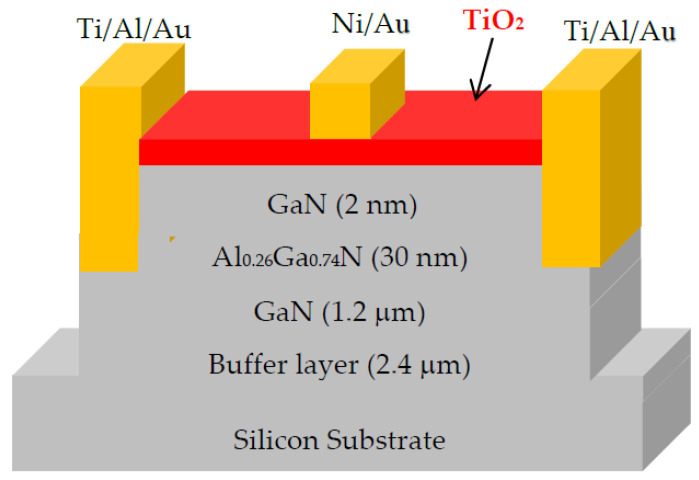
Schematic structure of TiO_2_ MOS-HEMT.

A TiO_2_ layer was deposited in the access region of the MOS-HEMT after the source and drain ohmic contacts had completely formed. TiO_2_ was deposited using an RF sputtering system. The TiO_2_ target was prepared from 99.99% TiO_2_ powder. The substrate temperature was 25 °C. The reactive gas was a mixture of Ar and O_2_ with fixed flow rates of 20 sccm and 5 sccm, respectively. Etchant (NH_4_OH/H_2_O_2_/H_2_O = 1:2:1) was used to remove TiO_2_ from the source and drain region. In order to improve the quality of the TiO_2_ layer and reduce the oxide trap, the as-deposited TiO_2_ was put into a rapid thermal annealing (RTA) system. The gate length and width were 1 μm and 100 μm, respectively. Figure 1 schematically depicts the TiO_2_ AlGaN/GaN MOS-HEMT.

## 3. Results

Hall data were measured for the studied HEMT without passivation [17]. Experimental results demonstrate that unannealed TiO_2_ passivation increases the 2-DEG concentration and reduces electron mobility. The increase in the 2-DEG concentration is caused by the trapping of positive charges at the surface, neutralizing the polarization charge [5,6]. The electron mobility of the device with the annealed TiO_2_ passivation is higher than that achieved using unannealed TiO_2_ passivation. Annealing increases electron mobility by reducing the traps.

**Figure 2:**
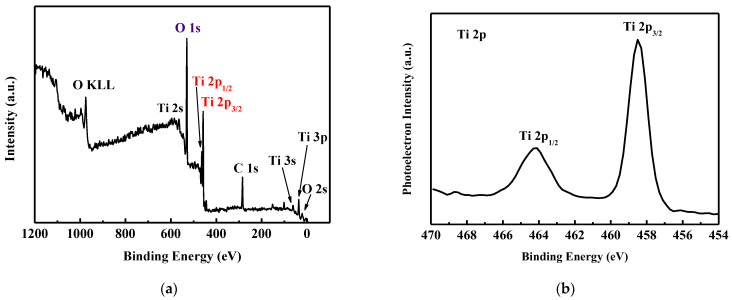
(**a**) XPS survey and (**b**) high-resolution XPS spectra of the as-deposited TiO_2_ films.

Figure 2a,b present typical XPS survey and high-resolution spectra of the as-deposited TiO_2_ films, respectively. Special attention should be paid to photoelectrons with binding energies between 454 eV and 470 eV. Figure 2b shows Ti 2p XPS data. The Ti 2p_2/3_ and Ti 2p_1/2_ spin-orbital splitting photoelectrons are located at around 458.5 eV and 464.2 eV, respectively, consistent with the values that are reported for TiO_2_ films [26,27,28].

**Figure 3:**
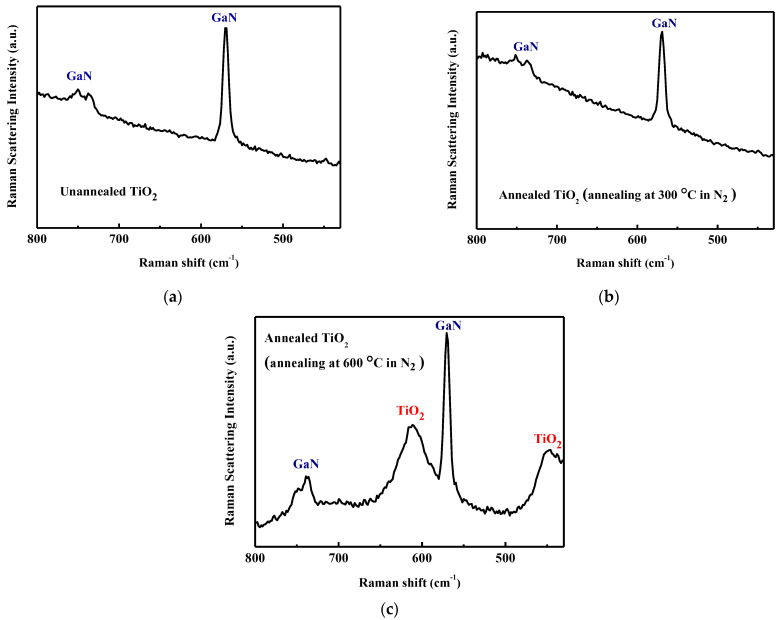
Raman spectra of (**a**) as-deposited, (**b**) 300 °C-annealed, and (**c**) 600 °C-annealed TiO_2_ on GaN.

Raman spectroscopy is a powerful diagnostic tool in the study of TiO_2_. Raman spectroscopy measurements are made. A TiO_2_ film is grown on GaN. The laser output power is set to 7.23 mW with an excitation wavelength of 532 nm. Figure 3a presents the Raman spectra of the as-deposited TiO_2_ taken at room temperature. Peaks that are associated with the GaN film are observed [29,30]. Figure 3b,c show the TiO_2_ films that are annealed at 300 °C and 600 °C, respectively. In general, crystalline TiO_2_ can exist in three phases, which are anatase, rutile, and brookite. Visible peaks due to the crystalline TiO_2_ phase are not obtained experimentally from the as-deposited TiO_2_ [Figure 3a] or 300 °C-annealed TiO_2_ [Figure 3b]. The 600 °C-annealed TiO_2_ yields two additional peaks [Figure 3c]. The emission bands at 447 and 610 cm^−1^ are identified as E_g_ and A_1g_ of the rutile structure, respectively [31,32,33]. Raman analysis shows that TiO_2_ remains amorphous when it is annealed at 300 °C but it adopts the rutile crystal structure when it is annealed at 600 °C. These Raman spectroscopic results are consistent with our previously reported X-ray diffraction (XRD) analysis [17].

**Figure 4:**
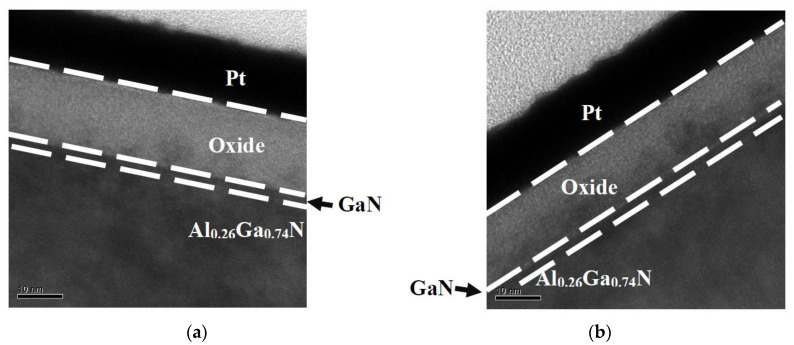
Cross-sectional transmission electron micrographs of studied heterostructure (**a**) without annealing and (**b**) following annealing at 300 °C in N_2_ ambient.

Transmission electron microscopy (TEM) samples are prepared using the focused ion beam (FIB) lift-out technique. Figure 4a,b display cross-sectional transmission electron micrographs of the TiO_2_/GaN/AlGaN heterostructure without and following annealing at 300 °C in an N_2_ ambient, respectively. The thickness of the 300 °C N_2_-annealed TiO_2_ film is about 14.7 nm. The thickness of the as-deposited TiO_2_ film is about 16.0 nm.

**Figure 5:**
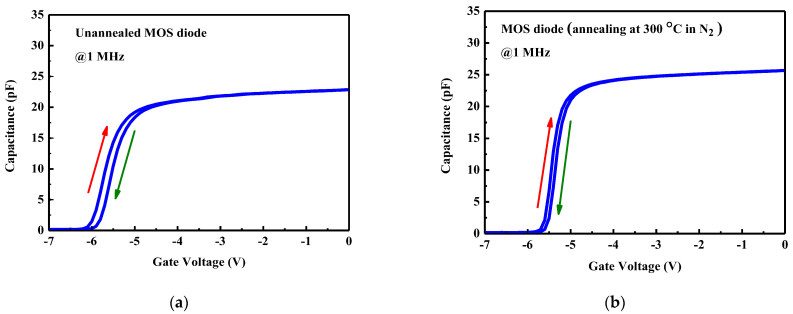
Capacitance–voltage hysteresis plots of (**a**) as-deposited and (**b**) 300 °C N_2_-annealed TiO_2_ MOS capacitors.

Gate oxide quality is evaluated using capacitance–voltage (C–V) measurements. The radius of the Schottky or MOS contact is 50.5 μm. Figure 5a,b are hysteresis plots of C–V data for the MOS capacitors without and following 300 °C annealing at 1 MHz, respectively. The C–V data are obtained by sweeping the gate voltage from zero to a negative value and back to zero. The 300 °C N_2_-annealed TiO_2_ MOS diode exhibits less hysteresis and a sharper transition from accumulation to depletion (or depletion to accumulation) than the unannealed MOS diode. Therefore, 300 °C N_2_ annealing improves the quality of the MOS diode [8,14,19,22,23]. From the zero-bias capacitance, the dielectric constant of the annealed TiO_2_ is calculated to be around 28.

**Figure 6:**
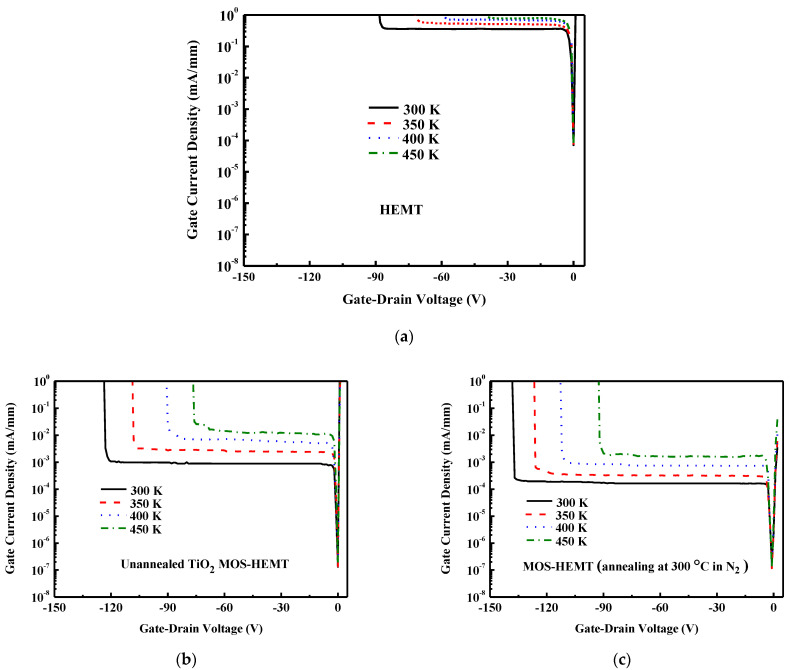
Two-terminal gate–drain breakdown plots for (**a**) HEMT, (**b**) unannealed MOS-HEMT, and (**c**) annealed MOS-HEMT at various temperatures.

Figure 6 plots the two-terminal gate-to-drain characteristics of the studied devices with a floating source that is operated at various temperatures. Figure 6b,c plot the results for the MOS-HEMT (without annealing) and MOS-HEMT (following N_2_ annealing at 300 °C), respectively. The breakdown voltage decreases as the temperature increases. Furthermore, the leakage current increases with temperatures. The 300 °C N_2_-annealed MOS-HEMT has a smaller leakage current than the unannealed MOS-HEMT under the same ambient condition because annealing reduces trap-assisted tunneling and Frenkel–Poole emission [34]. Experimental results clearly demonstrate the potential of our 300 °C N_2_-annealed MOS-HEMTs for high-temperature use.

**Figure 7:**
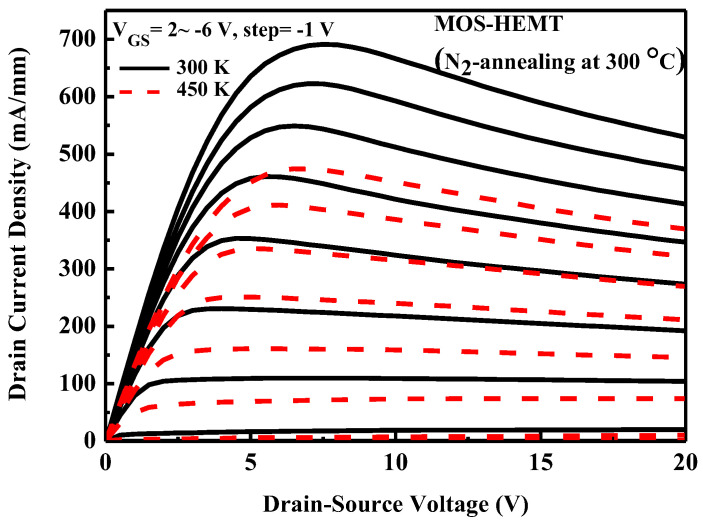
I_DS_–V_DS_ characteristics of MOS-HEMT at various temperatures.

**Figure 8:**
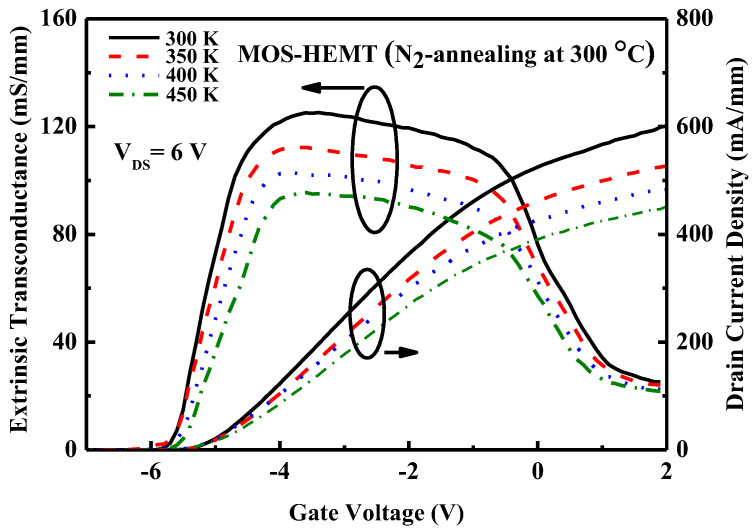
Drain current and extrinsic transconductance vs. gate-to-source voltage for studied devices at various temperatures.

Figure 7 plots the I_DS_–V_DS_ characteristics of the 300 °C N_2_-annealed MOS-HEMT. The data are measured at 300 K and 450 K. The gate voltage is swept from V_GS_ = +2 V to −6 V in −1 V steps. The drain current decreases as the temperature increases. Figure 8 plots the transfer characteristics and extrinsic transconductance of the 300 °C N_2_-annealed MOS-HEMT over a wide range of temperatures. The maximum extrinsic transconductane declines as the temperature increases. MOS-HEMTs operate stably at elevated temperatures up to 450 K with excellent pinch-off characteristics.

**Figure 9:**
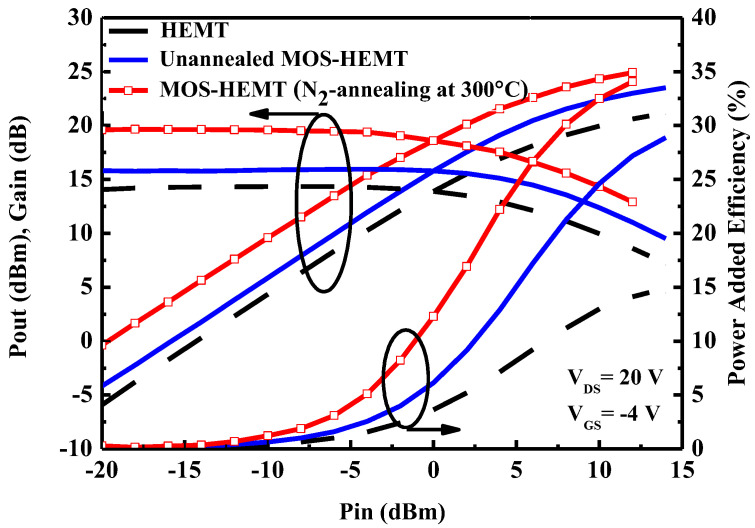
Load-pull data of the studied devices at 300 K. Each device is biased to V_DS_ = 20 V and V_GS_ = −4 V, and measurements are made at 2.4 GHz.

Figure 9 plots the measured CW power performance of the devices herein. Due to its lower gate leakage current, the maximum drain voltage of the MOS-HEMT could be higher than that of the HEMT, further favoring a high maximum rf power. The maximum output power (P*_out_*) and power-added efficiency (PAE) in Class A operation are given by [35].

(1) $$P_{out}=\frac{1}{8}I_{DS,\max}\left(V_{B}-V_{Dsat}\right)$$

(2) $$\mathrm{PAE}\;(\mathrm{in}\;\mathrm{percentage})=\frac{P_{out}(rf)-P_{in}(rf)}{P_{in}(dc)}\times 100\%$$

where *I_DS_*_,max_ is the maximum drain current, *V*_B_ is the breakdown voltage, *P_in_* (*dc*) is the dc power dissipation, *P_in_* (*rf*) is the input power, and *P_out_* (*rf*) is the output power. Table 1 compares the large-signal performances of the studied devices. The 300 °C N_2_-annealed MOS-HEMT exhibits the best power characteristics owing to the improved *I_DS_*_,max_, reduced leakage current, and improved breakdown voltage.

## 4. Conclusions

AlGaN/GaN MOS-HEMTs with TiO_2_ as the insulating layer are investigated. The effects of annealing treatment on the crystal structure of TiO_2_ are examined. The annealed TiO_2_/AlGaN/GaN MOS-HEMT exhibits the smallest leakage current and largest output power characteristics of the studied devices, and has great promise for 5G applications.

## Figures and Tables

**Table 1:** Comparison of 2.4 GHz large-signal performances of studied devices.

	P_OUT_ (dBm)	Gain (dB)	PAE (%)
HEMT	21.89	14.34	14.5
Unannealed MOS-HEMT	23.51	15.82	28.88
MOS-HEMT (N_2_ annealing at 300 °C)	24.92	19.65	34.08
